# Development of a health literacy framework for primary health care service users in Qatar: a mixed methods study

**DOI:** 10.1186/s12913-025-13919-8

**Published:** 2026-01-02

**Authors:** Mohamed Ahmed Syed, Ahmed Sameer Alnuaimi, Muslim Abbas Syed

**Affiliations:** Department of Clinical Research, Directorate of Clinical Affairs, Primary Health Care Corporation, Doha, Qatar

**Keywords:** Health literacy, Primary care, Socioecological model, Qatar

## Abstract

**Background:**

Health literacy (HL) has increasingly been recognized as a key factor influencing health outcomes and performance of health systems. Evidence suggests enhancing HL can improve access to care, self-management, and overall health outcomes. Despite Qatar’s well-established primary health care system, the country faces a high prevalence of non-communicable diseases. This study aimed to develop a health literacy framework tailored for primary health care service users (PHCSUs) in Qatar.

**Methods:**

A mixed methods study design which included a quantitative and qualitative component was employed. The quantitative component consisted of administering a validated Health Literacy Questionnaire (HLQ) to establish HL levels in PHCSUs. The qualitative component included interviews with PHCSUs and focus group discussion (FGD) with PHCC health care providers (HCPs). They were designed to identify preferred HL channels and gain an in-depth understanding of barriers and facilitators pertaining to HL strategies. The findings from the HLQ survey, HCP FGD and PHCSUs interviews along with strategies identified from literature were triangulated to develop a HL framework utilising the Socioecological Model structure as a foundation.

**Results:**

A total of 3124 HLQ survey responses were received. 26 HCPs and 16 PHCSUs participated in the FGD and interviews respectively. The study found HL in PHCSUs was influenced by expectations of HL, tailoring of messages, existing chronic conditions, language barriers, peer support, duration of clinical consultations, access to preventive services, regulation of health information sources, societal and cultural factors and community outreach.

**Conclusions:**

The study proposes a HL framework with HL determinants nested within the SEM in its entirety. It provides comprehensive view of HL and the many factors that influence it. The HL framework acknowledges the role of individuals and organizations, as well as the strong influence of overarching societal and cultural factors. This information can be utilized to devise interventions to improve HL through effective and innovative learning strategies. Implementation of these evidence-informed interventions and future research in this area can enhance patient centered care and improve the health outcomes.

**Supplementary Information:**

The online version contains supplementary material available at 10.1186/s12913-025-13919-8.

## Background

Health literacy (HL) emerged as a distinct concept in the 1970s, coinciding with a period in which health education was increasingly framed within the context of social policy [1]. In recent decades, there has been an increased interest in the concept driven by its demonstrable impact on individual and population health outcomes, and its potential to enhance sustainability and resilience of healthcare systems [2, 3].

In recent years, numerous multidimensional perspectives and instruments measuring HL have been developed and reported in literature [1, 4, 5]. The concept at an individual level can be categorized as functional HL (basic skills including reading and writing skills), communicative or interactive HL (advanced skills that allows individuals to extract information and their related meanings from multiple communication channels and put them into practice) and critical HL (advanced skills that are needed to analyze information and use knowledge in order to apply more control on life events and situations) [2, 6]. At a health care organization level, HL can be described as enabling people to navigate, understand, and apply information and services to take care of their health [7]. This approach entails the design of health services that are accessible and user-friendly, promote health and prevent disease, advance equity, and develop a responsive health care system that empowers individuals to make informed decisions regarding their health [8].

Sørensen et al. undertook a systematic review to develop an integrated definition capturing the most comprehensive evidence-based dimensions of HL [1]. According to their definition, HL “is linked to literacy and entails people’s knowledge, motivation and competences to access, understand, appraise, and apply health information in order to make judgments and take decisions in everyday life concerning healthcare, disease prevention and health promotion to maintain or improve quality of life during the life course” [1].

Fundamental principles encompassed within HL empower individuals in making informed decisions about their health promoting patient centered care [9–11]. HL has increasingly been recognized as a key factor influencing health outcomes and performance of health systems [12]. Evidence suggests that higher HL levels can significantly contribute towards accessibility to health care services, adherence to treatment, better understanding of consent-to-treat forms and general health, early disease detection and enhanced capability to effectively utilize the different health information channels within operating health systems [6, 13]. On the contrary, lower HL levels are associated with lower utilization of health screening, poor health behaviour and increased hospitalization rates leading to increase economic burden on health systems [14–19]. Globally, low HL is a concern due to its significant public health implications. Studies report that nearly 50% of individuals encounter difficulties managing health problems due to inadequate HL skills [18].

The Socioecological Model (SEM) is an integrated framework that examines how factors at different levels of influence health related behaviours [20–22]. The various levels within SEM include intrapersonal (individuals’ characteristics, including knowledge, attitudes, and behaviors), interpersonal level (individuals’ familial and social networks and its impact on healthcare practices and contribution to different experiences), institutional (encompassing operations of social institutions, including health facilities and their health care professionals and its role in health care decision-making), community-level (entails the social and physical environment that includes the greater community) and policy level (utilization and access to healthcare services, and the adoption of healthy behaviors) [23]. The SEM demonstrates that individual behaviour is shaped by factors at multiple levels [20–22]. Hence it is relevant to HL and has been used as a framework to study HL in various contexts [7, 24–27].

Primary health care plays a crucial role in HL as the first point of contact for health care needs in terms of early disease detection, prevention and controlling the burden of disease (particularly non-communicable diseases) in the general population [28]. Literature indicates it is important to identify HL gaps and challenges within primary care settings to build locally fit-for-purpose interventions that are acceptable, adaptable and accessible [29, 30]. These interventions can be implemented by active community engagement achieved by local initiatives and ownership [31]. Moreover, it is worthwhile exploring how social and cultural determinants of health impact HL among service users accessing primary care services. Furthermore, studying HL within a population can serve as a lens for implementing effective treatment modalities pertaining to non-communicable diseases and to better understand and design health promotion strategies for general wellbeing specifically within the domains of preventive medicine [32].

Qatar is a sovereign and independent country in the Middle East. Over the recent years, it been significantly investing in its publicly funded healthcare system delivered by the Primary Health Care Corporation (PHCC – for primary health care services) and Hamad Medical Corporation (HMC- for secondary and tertiary care services). As with most countries globally, Qatar faces a high burden of non-communicable diseases [33, 34]. Enhancing HL across its primary health care population can lead to improved access to healthcare, more efficient use of services, better self-care and disease management, and ultimately, more positive health outcomes. This study aimed to develop a HL framework tailored for primary health care service users (PHCSUs) in Qatar. The framework is intended to guide design and implementation of locally relevant HL related interventions that are acceptable, adaptable, and accessible.

## Methods

A detailed protocol outlining the methods employed for the study has been published [35].

### Study settings and design

The study was conducted at PHCC which operates a family medicine model [36]. Its services include Family Medicine, Women’s Health, Child and Adolescent Health, Mental Health, Dental and Oral Health, Wellness, Chronic Disease Screening, Pharmacy, Laboratory, Radiology, Home Healthcare etc. across its 31 primary health care centers — ensuring continuity and comprehensive care for all patients. Each primary health care center is equipped with modern healthcare facilities managed by multidisciplinary teams (MDT) delivering the highest standards of primary healthcare services [37, 38].

A mixed methods study design was employed. It included a quantitative and qualitative component. The quantitative component consisted of administering a validated online Health Literacy Questionnaire (HLQ) to establish HL levels of the PHCC service [39]. The qualitative component included individual semi-structured interviews with PHCSUs and a focus group discussion (FGD) with PHCC health care providers (HCPs) to identify preferred HL channels and gain an in-depth understanding of barriers and facilitators pertaining to HL strategies. The data from the quantitative and qualitative components were triangulated to develop a HL framework.

### Recruitment and data collection

#### Quantitative component

HL was evaluated using a validated HLQ tool to provide a multi-dimensional understanding of HL strengths and weaknesses [39]. It was administered in English and Arabic and included 44 items across nine domains (Table S1 supplementary file).

Adult PHCSUs (18 years and above) with a valid mobile phone number were recruited using a stratified random sampling (not proportional to size) from the total PHCC registered population. The response rate was expected to be 1%. A sample size of 3000 was required to calculate a prevalence estimate for the overall population of 50% with a margin of error < 2% at 95% confidence level. Therefore, a total of 300,000 individuals were invited to participate using Short Message Service (SMS) with a link to the HLQ tool.

#### Qualitative component

Interviews with 16 PHCSUs were conducted between 04/12/2024 to 05/12/2024. Adult PHCSUs (18 years and above) visiting a PHCC health centre during these dates were randomly approached by MAS (author 3) and invited to participate in the study. The purpose of the study was explained and if the PHCSU agreed to participate, they were invited to a private room. Written informed consent was taken from PHCSUs before starting the interview. Each interview lasted between 45 and 60 min. The interviews were semi-structured utilising an interview guide and script. The interviews were recorded with the permission of PHCSUs. Once the interview had taken place all the data collected was anonymised.

HCPs with at least one year of work experience at PHCC were recruited using a non-probability convenience sampling with the help of health centre managers. Prior to agreeing, HCPs were provided with a participant information leaflet and FGD topic guide (Appendix 1 supplementary file).

One FGD was held on 24/02/2025 in which 26 HCPs participated. At the start of the FGD, HCPs were advised that their participation was voluntary and verbal consent was recorded. The FGD discussion was facilitated using the FGD guide and audio recorded. The main themes of the discussions were derived from the broader themes outlined in literature pertaining to domains of HL, associated channels and existing challenges and gaps in its implementation within primary care settings [40–45]. 

### Data analysis

#### Quantitative component

In the HLQ tool, each domain comprises four to six items. The first five domains (HLQ part 1) were scored using response options indicating the level of agreement, ranging from strongly disagree (1) to strongly agree (4). The last four domains (HLQ part 2) reported on capabilities, scored from “cannot do or always difficult” (1) to “always easy” (5). The domain scores were calculated as an average of the item scores with higher scores indicating better HL levels. A high level HL for the five HLQ Part 1 domains was defined as a score greater than 3, while a high level HL for the four HLQ Part 2 domains was defined as a score greater than 4. Frequencies, proportions and confidence intervals were calculated by domain (Table S1 supplementary file).

#### Qualitative component

The qualitative data once recorded was transcribed verbatim, and then analyzed using thematic analysis [46]. This approach encompasses ‘interpreting, exploring, and reporting patterns and clusters of meaning within the given data’ [47] and was facilitated by reading and re-reading the transcripts for a full familiarization. This was followed by application of open codes to four transcripts to identify emerging themes of relevance by MAS (author 3) and ASA (author 2) [48]. A Computer Assisted Qualitative Data Analysis (CAQDAS) package (NVivo 12 for Windows) was utilized for this process. This was followed by agreement between MAS (author 3) and ASA (author 2) on a set of codes to be used with the rest of the transcripts. During this stage categories were constructed and defined by grouping of codes. This led to the development of a working code framework which was utilized with the rest of the data and amended as necessary. The study reports results in accordance with ‘Consolidation criteria for reporting qualitative research (COREQ) and ‘Standards for reporting qualitative research ‘(SRQR) guidelines [49, 50]. 

### Development of a health literacy framework

The study utilised the SEM levels as a foundation for the HL framework for PHCSUs in Qatar. The findings from the HLQ survey, HCP FGD and PHCSUs interviews along with strategies identified from literature [51–66] were triangulated to develop a HL framework by categorising them to an appropriate SEM level.

## Results

A total of 3124 HLQ survey responses were received (Arabic = 1420 and English = 1704). 26 HCPs and 16 PHCSUs participated in the FGD and interviews respectively (Table 1). PHCSUs age ranged between 20 and 65 years. 44% (*n* = 7) PHCSUs had co-morbidities and reported polypharmacy. A broad range of HCPs participated in the FGD - Radiologists, physicians, psychiatrist, dietitians, health educators, nurses, lab technologists, and pharmacists (Table 2). Majority of them had significant years of experience.

**Fig. 1:**
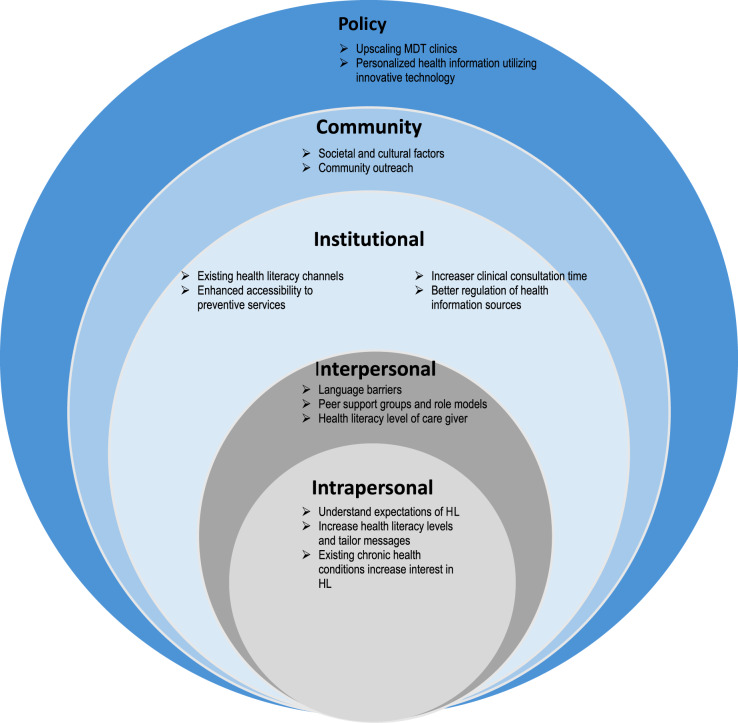
Health literacy framework for primary health care service users in Qatar

After data analysis, results were catergorised to relevant SEM framework levels (i) intrapersonal (ii) interpersonal (iii) institutional (iv) community and (v) policy (Fig. 1).

**Table 1:** Demographic details of service users who participated in interviews (*n* = 16)

Participant code	Gender	Age group	Polypharmacy & co-morbidities*
PID-01	Male	30–35 years of age	-
PID-02	Male	40–45 years of age	+
PID-03	Female	25–30 years of age	-
PID-04	Male	40–45 years of age	-
PID-05	Male	55–60 years of age	+
PID-06	Female	45–50 years of age	-
PID-07	Female	50–55 years of age	+
PID-08	Male	30–35 years of age	-
PID-09	Male	25–30 years of age	-
PID-10	Female	20–25 years of age	+
PID-11	Male	35–40 years of age	-
PID-12	Male	30–35 years of age	-
PID-13	Female	45–50 years of age	+
PID-14	Female	25–30 years of age	-
PID-15	Male	45–50 years of age	+
PID-16	Male	60–65 years of age	+

*(+ having issues of polypharmacy and co-morbidities, - not having issues)

**Table 2:** Demographic details of healthcare professionals who participated in FGD (*n* = 26)

Participant no & job role of the participant	Years of experience	Gender
PT 1: Consultant Radiologist	10–15 years	Male
PT2: Specialist Otolaryngologist	15–20 years	Male
PT3: Charge Nurse	15–20 years	Female
PT4: Pharmacist-lead	20–25 years	Male
PT5: Senior Consultant Pediatrician	20–25 years	Female
PT6: Senior Consultant Child and Adolescent Psychiatry	5–10 years	Female
PT7: Specialist Family Medicine	20–25 years	Male
PT8: Health Educator	15–20 years	Male
PT9: Health Educator	5–10 years	Female
PT10: GP Dentist-Lead	15–20 years	Female
PT11: Lab Technologist	10–15 years	Male
PT12: Consultant Community Medicine	15–20 years	Female
PT13: Charge Nurse	15–20 years	Female
PT14: Health Educator	5–10 years	Male
PT15: Pharmacist	Less than 5 years	Female
PT16: Clinical Pharmacist	15–20 years	Male
PT17: Dietician	20–25 years	Male
PT18: Dietician	5–10 years	Male
PT19: Health Educator	Less than 5 years	Female
PT20: Dietician	5–10 years	Male
PT21: Pharmacist	15–20 years	Male
PT22: Nurse	Less than 5 years	Female
PT23: Nurse	15–20 years	Female
PT24: Radiology Technologist	10–15 years	Male
PT25: Consultant Family Physician	20–25 years	Female
PT26: Senior Pharmacist	15–20 years	Male

### Intrapersonal factors

Participants reported various intrapersonal factors that influence HL. These encompass factors that are intrinsic such as HL levels, utility of the concept and influence of current health condition. HL levels and utility of the concept were most frequently discussed by PHCSUs. Intrapersonal factors reported by the participants were influenced by determinants across all other SEM levels, especially those relating to HL levels of care givers, peer support groups or social networks.

#### Expectations of HL

HCPs defined the concept of HL as the extent to which PHCSUs can comprehend and utilize health information to make informed health related decisions for themselves and others. PHCSUs defined the concept of HL and its utility as acquisition of knowledge to adopt healthy lifestyles and self-manage diseases. For instance, a female service user commented while defining HL:

Health literacy is acquiring knowledge to take care of my health, manage my disease and make healthy lifestyle choices [PID3]

#### Health literacy levels

HCPs highlighted the significance of determining HL levels amongst PHCSUs to tailor health information communicated to them. A nurse commented on the significance this to tailor the health information provided:

It is important to understand HL levels of patients when we interact with them. The information and interaction can be tailored to how they respond, what they might be expecting, how effectively we communicate. Taking this into consideration is very important [PT23]

PHCSUs mentioned HL levels as the key limitation in interpreting and utilizing the health information provided and available to them. This was attributed to the diverse multi-lingual expat population residing and accessing health services in the country.

The HLQ survey (*n* = 3124) found that less than half of the respondents demonstrated a high level of HL across the nine HLQ domains (Table S2 supplementary file). The high-level construct rates ranged between 27.5% and 42.7%. The highest rates were observed in Domain 9 (who could comprehend all written information in relation to their health and able to write appropriately on forms where required) and in Domain 3 (Recognize the importance and are able to take responsibility for their own health), 42.7% and 40.1% respectively. Conversely, lowest rates (27.5%) were observed in Domain 7 (able to find out about services and supports so they get all their needs met. Able to advocate on their own behalf at the system and service level). Arabic speaking HLQ participants consistently showed higher rates of high-level HL across all domain constructs compared to English speaking HLQ participants.

#### Influence of chronic conditions

PHCSUs highlighted that individuals living with chronic disease conditions were more likely to be updated and receptive to health information. The following excerpts present opinions on the influence of chronic health conditions on PHCSUs HL levels.

I am diabetic, and I try to keep myself informed and updated about my condition [PID16]

I think anyone with a condition for a long period is more likely to be interested and informed about it [PID13]

### Interpersonal factors

Interpersonal factors highlighted by participants included language barriers, HL levels of caregivers, significance of peer support groups and social networks and motivation by influential role models or celebrities.

#### Language barriers

PHCSUs reported language barriers as a key limitation in interpreting and utilizing the health information provided and available to them. They said this could be attributed to the diverse multilingual population residing in the country. A male service user commented:

I believe it is the language barrier, that is what can be a challenge. There are people working in health services from abroad who can speak different languages. They can help overcome this barrier if they are appropriately paired or translators made available. [PID1]

#### Health literacy levels of caregivers

PHCSUs highlighted the importance and impact of HL of caregivers on disease management and that its influence on HL of patients and their health outcomes is significant. PHCSUs commented:

Involving the family in health education to provide care for patients can be good strategy [PT8]

The caregiver, particularly the family, plays an important role in taking care of patients. If caregivers can be trained or be provided with additional information, they can help improve health literacy levels of patients and their health outcomes [PT3]

#### Peers support groups and role models

PHCSUs emphasized the role of peer support groups and motivation by influential role models as effective strategies to meet the needs of people particularly those living with chronic diseases. They highlighted that people living with chronic diseases can be motivated by a celebrity or role model living with a similar health condition by imparting relevant health information. For example, a South Asian service user said:

I have seen on TV that an Indian actor she had cancer, and she fought it. She survived and then she has become a role model in motivating other people by letting them know how the disease can be fought and how one can survive [PID15]

### Institutional factors

Institutional factors discussed by participants included HL channels, accessibility of health services and duration of clinical consultations.

#### Health literacy channels

Participants discussed existing HL channels utilised by PHCC and their impact on the HL of PHCSUs. In context of the PHCC, participants reported a range of HL channels currently being utilized which were mainly social media platforms (Instagram etc.), print media, online search engines, SMS and online health reports. A male Asian PHCSU emphasized the effectiveness of SMS in keeping PHCSU informed and updated about their health condition:

As per my experience, we receive a lot of text messages, especially if you are patient. PHCC follows up with good messages. SMS or different messages through emails, especially for the patient would be beneficial [PID9]

HCPs reported that SMS were frequently used to follow up with PHCSUs and share relevant health information. The availability of health educators within the primary care services to improve HL was also highlighted. Moreover, a community medicine consultant mentioned use of mobile phone applications tailored to address the health information needs of service users:

There was a pilot study that was done in 2019 for a mobile phone application. PHCSUs downloaded the application, and he or she could contact a MDT team anytime if she or he had a health problem [PT12]

HCPs recommended novel use of everyday gadgets such as mobile phones and distribution of printed summarized information related to medications, laboratory tests, and key points from clinical consultations were also suggested.

PHCSUs highlighted prioritizing interactive sessions. The following excerpts capture the suggestions by service users of engaging in interactive channels in receiving health information.

Now these days are a lot of events relating to activities, mostly its boring lectures and lengthy information which is hard to listen to and follow. If we can have events or activites, which are not a one-way like seminars and symposiums were you just listen and can’t interact, it will be helpful [PID4]

I attended an event where they provided virtual advice using a device to check all the parts of the body and how they are affected. Such interactive sessions can be beneficial, and should be implemented more to improve HL of service users [PID7]

People can remember and relate to more when they see things visually. I believe if something like a drama/skit is arranged in a community setting where people can see and relate to, they would understand and remember information better [PID13]

#### Duration of clinical consultations and access to preventive services

PHCSUs described PHCC HCPs as engaging, empathetic and provide relevant information during clinical consultations. While HCPs stated that they experience time constraints during clinical consultations. Furthermore, PHCSUs highlight difficulties accessing preventive services. These factors were considered barriers in disseminating health information to PHCSUs. In context of accessibility a health educator stated:

Despite numerous preventive health services being available, PHCSUs state they experience difficulties in accessing them. [PT8]

#### Regulating health information sources

The internet provides access to a vast amount of information; its trustworthiness is a growing concern. The volume of information available can make it challenging to discern reliable sources from misinformation or biased content. HCPs expressed a need to regulate publicly available health information sources. A consultant pediatrician commented:

Social media can be utilized to provide information. However, it should be regulated by the Ministry of Public Health. Furthermore, we should educate the public to access information and updates from trusted sources [PT3]

### Community factors

Community factors were considered social and cultural determinants that influence HL. The significance of community outreach programs was discussed.

#### Influence of social and cultural determinants of health

PHCSUs highlighted the influence of social and cultural determinants of health and how they influence attitudes and perceptions towards receiving and implementing relevant health information. The following excerpts summarise these discussions.

It has been a few months since I moved to Qatar from Canada. Back home everyone is conscious about their health and always looking up information online. People engage actively and so did I with my doctor during consultation. I think the overall belief and attitude of a society and community can influence the effectiveness and use of available health information [PID14]

Some elders in my family pay little attention to a healthy lifestyle and getting more information about it. Maybe it is a cultural perception in Arabs [PID16]

Personally, I think medication that is offered is worse than the disease itself. This can be prevented; In Europe and America doctors prescribe fewer medications and focus more on awareness [PID1]

#### Community outreach programs

HCPs note that health information shared with PHCSUs may not be understandable to everyone. Hence, they recommend having community outreach programs via community health champions. The champions could act as a bridge between communities and health services, often facilitating access to information, resources, and support. They could leverage their community knowledge and connections to promote health and well-being within their local area.

A lot of people don’t know how to read or understand information. They ask other people. However, those people may not be the right people to help them. A community health champion could support them with their community knowledge and connections to promote health, particularly if the information is complex. [PID1]

#### Policy factors

Participants suggested upscaling the already existing MDT clinics to provide comprehensive health information (aimed at increasing their HL levels about disease management and lifestyle behaviors) with due consideration of people living with chronic diseases. A community medicine consultant suggested:

Currently there are MDT clinics to support chronic disease patients which include pharmacists, doctors and nurses, with referrals to dietitians. Further developing such clinics will provide more health information to PHCSUs and increase their health literacy levels leading to desired health outcomes through compliance with treatment and improving self-care [PT12]

Moreover, suggestions were provided by participants to prioritize and further implement personalized health information utilizing innovative technologies such as artificial intelligence (AI). The following excerpts highlight suggestions about further exploring the role of personalized health information in increasing HL:

An individual who is diagnosed with a chronic condition, for example diabetes, needs to be sent constant reminders about appointments to ensure they are not missed. PHCC has all our numbers, so the most obvious route will be sending SMS to every diabetic patient. Clinics need to be sent those reminders to get the blood test done. Sporadically, sending information about diet and nutrition can also be helpful [PID11]

The possibility of using AI to send personal health information focusing on their own health issues, this is the future, and it will really benefit patients in managing their disease [PID7]

## Discussion

### Main findings of the study

Globally, countries are making efforts towards improving HL rates, however they are falling short of achieving sufficient results [67]. The study developed a HL model for PHCSUs in Qatar. It reports key factors associated with HL at each level of SEM that influence HL. In general, the study found that HL among PHCSUs was influenced by expectations of HL, tailoring of messages, existing chronic conditions, language barriers, peer support, duration of clinical consultation, access to preventive services, regulation of health information sources, societal and cultural factors and community outreach.

PHCSUs mainly associate expectations of HL with acquisition of knowledge to adopt healthy lifestyles and navigate personal health for disease prevention. HCPs described the level of HL as a benchmark which directly influences PHCSUs’ comprehension and utilization of health information to make health-related informed decisions. HCPs reported SMS as one of the main HL channels. Arabic speaking HLQ participants consistently showed higher rates across nine HL domain constructs compared to English speaking HLQ participants.

### Comparison of the key findings of the study with existing literature

The study identified language barriers (intepersonal level) and sociocultural background (community level factor) within the SEM as a limiting factor in active transfer, comprehension and utlization of relevant health information to navigate health care services, self management of health condition and overall general well being. The impact of cultural and linguistic barriers in HL and its association with poorer health outcomes are well documented in recent literature [68–70].

At the intrapersonal level, the study found PHCSUs who responded to the Arabic language HLQ demonstrated a higher rates of high-level HL across all domains encompassed within the tool. This finding suggests they have a better understanding of navigating the health service, accessing essential health services, managing their disease condition, comprehending health information transferred through available HL channels and ability to seek support from HCPs when compared to PHCSUs who responded to English language HLQ. Studies [69, 71–74] substantiate this finding which indicates that native service users have higher rates of accessbility to the primary health care services, achieve desired health outcomes and report lesser unmet health care needs in comparsion with immigrant population. It can be argued that native service users might have higher level of language proficiency of the locally spoken language in the country and increased familarity and sense of ownsership while navigating, analyzing and utilzing HL channels to access primary health care services [72]. 

At the institutional level, PHCSUs proposed interactive sessions while designing HL interventions. These suggestions are supported by existing evidence which substantiate the fact that interactive HL training interventions for HCPs working in primary care settings improves their knowledge, approach, and confidence in using HL strategies with service users and families accessing the service [60]. 

At the policy level, PHCC has invested significantly in improving the HL for patients with chronic conditions by establishing MDTs. The significance of upscaling the already existing MDTs, increasing the HL levels of caregivers and further implementing personalized health information utilizing innovative technologies such as AI to provide tailored health information was emphasized by HCPs. This strategy was perceived by HCPs to improve the HL levels of PHCSUs and address their unmet health information needs particularly among people living with chronic conditions. Recent literature also suggests that integrative models of care composed of MDTs can increase quality of care, patient satisfaction, HL levels and can lead to better chronic condition management. Moreover, evidence also supports that increased HL of caregivers can promote efficient utilization of the health care services [54, 75]. Furthermore, recent literature also emphasises the need for further utilization of personalized health information using AI to improve the HL levels of service users [61, 62]. 

### Strengths and limitations of the study

The mixed methods study design enabled triangulation of contextualized insights into individual experiences and perspectives to gain a more nuanced understanding of HL related factors. The HL framework was developed using SEM levels which is widely recognised in existing literature. While it provides a holistic view, it also provides information by levels which can be conisdered seperately by decision makers to implement improvement. Study participants had diverse sociodemographic characteristics representing the overall general population of Qatar. This ensured that HL related factors captured by the study were comprehensive and represented the diverse multicultural population residing in Qatar. Moreover, the study also utilized HLQ, which is a validated and comprehensively used tool to measure HL levels in populations. The study, before study initiation, factored non-response rates when calculating the study sample size. However, this was underestimated. Another limitation is that the HLQ adminstered did not collect socio-demographic details of participants. Therefore the HL levels could not be analysed and reported by socio-demographic sub catergories.

## Conclusion

The study proposes a HL framework with HL determinants nested within the SEM in its entirety. It provides comprehensive view of HL and the many factors that influence it. The HL framework acknowledges the role of individuals and organizations, as well as the strong influence of overarching societal and cultural factors. This information can be utilized to devise interventions to improve HL through effective and innovative learning strategies. Implementation of these evidence-informed interventions and future research in this area can enhance patient centered care and improve the health outcomes.

## Supplementary Information

Below is the link to the electronic supplementary material.

Supplementary Material 1

## Data Availability

All data generated or analysed during this study are included in this published article and its supplementary information files.

## References

[1] Sørensen K, et al. Health literacy and public health: A systematic review and integration of definitions and models. BMC Public Health. 2012;12(1):80. 22276600 10.1186/1471-2458-12-80 PMC3292515

[2] Nutbeam D. Health literacy as a public health goal: a challenge for contemporary health education and communication strategies into the 21st century. Health Promot Int. 2000;15(3):259–67.

[3] Peerson A, Saunders M. Health literacy revisited: what do we mean and why does it matter? Health Promot Int. 2009;24(3):285–96. 19372101 10.1093/heapro/dap014

[4] Liu C et al. What is the meaning of health literacy? A systematic review and qualitative synthesis. Fam Med Community Health. 2020;8(2). 10.1136/fmch-2020-000351 PMC7239702 32414834

[5] Pinheiro P. Conceptualizations of health literacy: past Developments, current Trends, and possible ways forward toward social practice. Health Lit Res Pract. 2021;5(2):e91–5. 34213999 10.3928/24748307-20210316-01 PMC8082953

[6] Nutbeam D. The evolving concept of health literacy. Soc Sci Med. 2008;67(12):2072–8. 18952344 10.1016/j.socscimed.2008.09.050

[7] Farmanova E, Bonneville L, Bouchard L. Organizational health literacy: review of Theories, Frameworks, Guides, and implementation issues. Inquiry. 2018;55:46958018757848. 29569968 10.1177/0046958018757848 PMC5871044

[8] Brach C, et al. Ten attributes of health literate health care organizations. NAM Perspectives; 2012.

[9] Organization WH. Health literacy development for the prevention and control of noncommunicable diseases. Volume 4: Case studies from WHO National Health Literacy Demonstration Projects. World Health Organization; 2022.

[10] Pelikan JM. Health-literate healthcare organisations. In International Handbook of Health Literacy. Policy Press; 2019. pp. 539–54.

[11] Drapkina O, et al. The WHO European action network on health literacy for prevention and control of noncommunicable diseases. Public Health Panorama. 2019;5(2–3):197–200.

[12] Pleasant A. Advancing health literacy measurement: a pathway to better health and health system performance. J Health Communication. 2014;19(12):1481–96. 25491583 10.1080/10810730.2014.954083 PMC4292229

[13] Kindig DA, Panzer AM, Nielsen-Bohlman L. Health literacy: a prescription to end confusion*.* 2004. 25009856

[14] DeWalt DA, et al. Literacy and health outcomes: a systematic review of the literature. J Gen Intern Med. 2004;19:1228–39. 15610334 10.1111/j.1525-1497.2004.40153.x PMC1492599

[15] Berkman N, et al. Literacy and health outcomes: evidence report/technology assessment. Rockville: Agency for Healthcare Research and Quality; 2004. Publication No.: pp. 04-E007.

[16] Pink B. Health Literacy, Australia. Canberra: Australian Bureau Statistics; 2006.

[17] Fleary SA, Joseph P, Pappagianopoulos JE. Adolescent health literacy and health behaviors: A systematic review. J Adolesc. 2018;62:116–27. 29179126 10.1016/j.adolescence.2017.11.010

[18] Berkman ND, et al. Low health literacy and health outcomes: an updated systematic review. Ann Intern Med. 2011;155(2):97–107. 21768583 10.7326/0003-4819-155-2-201107190-00005

[19] Eichler K, Wieser S, Brügger U. The costs of limited health literacy: a systematic review. Int J Public Health. 2009;54(5):313–24. 19644651 10.1007/s00038-009-0058-2 PMC3785182

[20] Bronfenbrenner U. Toward an experimental ecology of human development. Am Psychol. 1977;32(7):513.

[21] McLeroy KR, et al. An ecological perspective on health promotion programs. Health Educ Q. 1988;15(4):351–77. 3068205 10.1177/109019818801500401

[22] Sallis JF, Owen N, Fisher E. Ecological models of health behavior*.* Health behavior: theory, research, and practice. 5th ed., 2015. p. 43–64.

[23] Olaniyan A, Isiguzo C, Hawk M. The socioecological model as a framework for exploring factors influencing childhood immunization uptake in Lagos state, Nigeria. BMC Public Health. 2021;21(1):867. 33952252 10.1186/s12889-021-10922-6 PMC8098781

[24] Fenta ET, et al. Exploring barriers of health literacy on non-communicable disease prevention and care among patients in North Wollo zone public hospitals; Northeast, Ethiopia, 2023: application of socio-ecological model. BMC Public Health. 2024;24(1):971. 38581006 10.1186/s12889-024-18524-8 PMC10998356

[25] Yin M, Yangyuen S, Somdee T. The relation of Social-ecological factors and health literacy to medical students’ alcohol use behavior in Hubei Province, China. J Res Health Sci. 2023;23(4):e00599. 38315914 10.34172/jrhs.2023.134 PMC10843315

[26] Luo P, et al. Exploring the factors influencing nutritional literacy based on the socioecological model among patients with age-related macular degeneration: a qualitative study from China. BMJ Open. 2024;14(5):e081468. 38806439 10.1136/bmjopen-2023-081468 PMC11138290

[27] Dadaczynski K, et al. Editorial: the Social-Ecological context of health literacy. Front Public Health. 2022;10:897717. 35558540 10.3389/fpubh.2022.897717 PMC9087034

[28] Organization WH. Health literacy development for the prevention and control of noncommunicable diseases. Volume 2: A globally relevant perspective. World Health Organization; 2022.

[29] Zarcadoolas C, Pleasant A, Greer DS. Advancing health literacy: a framework for understanding and action. Wiley; 2006.

[30] Williams MV, et al. Relationship of functional health literacy to patients’ knowledge of their chronic disease: a study of patients with hypertension and diabetes. Arch Intern Med. 1998;158(2):166–72. 9448555 10.1001/archinte.158.2.166

[31] Osborne RH, et al. Health literacy development is central to the prevention and control of non-communicable diseases. BMJ Global Health. 2022;7(12):e010362. 36460323 10.1136/bmjgh-2022-010362 PMC9723891

[32] Vamos S, Rootman I. Health literacy as a lens for understanding non-communicable diseases and health promotion. In Global handbook on noncommunicable diseases and health promotion. Springer; 2013. pp. 169–87.

[33] Syed MA, et al. Prevalence of non-communicable diseases by age, gender and nationality in publicly funded primary care settings in Qatar. BMJ Nutr Prev Health. 2019;2(1):20–9. 33235953 10.1136/bmjnph-2018-000014 PMC7678476

[34] Syed MA, et al. Prevalence of metabolic syndrome in primary health settings in qatar: a cross sectional study. BMC Public Health. 2020;20(1):611. 32362284 10.1186/s12889-020-08609-5 PMC7196222

[35] Syed MA, Alnuaimi AS, Syed MA. Exploring health literacy pertaining to general wellbeing and chronic disease management among population registered within primary healthcare system: A study protocol. PLoS ONE. 2025;20(10):e0333194. 41056344 10.1371/journal.pone.0333194 PMC12503302

[36] Syed MA, et al. Development of a model to deliver primary health care in Qatar. Integr Healthc J. 2020;2(1):e000040. 37441307 10.1136/ihj-2020-000040 PMC10327457

[37] Syed MA, Razaq S, Alnuaimi AS. Epidemiological investigation of disease Patterns, Accessibility, and patient characteristics following the introduction of dermatology specialty clinics within primary care settings in Qatar. Cureus. 2024;16(11):e72964. 39634977 10.7759/cureus.72964 PMC11615832

[38] Syed MA, et al. Key service delivery processes, challenges and barriers to healthcare access for managing diabetes outside target HbA1c levels in primary care settings in Qatar: a qualitative inquiry of healthcare professionals’ and service users’ perspectives. BMJ Public Health. 2025;3(1). 10.1136/bmjph-2024-001969 PMC12107455 40433075

[39] Osborne RH, et al. The grounded psychometric development and initial validation of the health literacy questionnaire (HLQ). BMC Public Health. 2013;13(1):658. 23855504 10.1186/1471-2458-13-658 PMC3718659

[40] Hersh L, Salzman B, Snyderman D. Health literacy in primary care practice. Am Family Phys. 2015;92(2):118–24. 26176370

[41] Mor-Anavy S, Lev-Ari S, Levin-Zamir D. Health literacy, primary care health care providers, and communication. Volume 5. HLRP: Health Literacy Research and Practice; 2021. pp. e194–200. 3. 10.3928/24748307-20210529-01 PMC8279021 34260319

[42] Skidmore N, et al. Acceptability and feasibility of virtual reality to promote health literacy in primary care from the health professional’s view: A qualitative study. Patient Educ Couns. 2024;123:108179. 38367303 10.1016/j.pec.2024.108179

[43] İlhan N, et al. Health literacy and diabetes self-care in individuals with type 2 diabetes in Turkey. Prim Care Diabetes. 2021;15(1):74–9. 32646764 10.1016/j.pcd.2020.06.009

[44] Shahid R, et al. Impact of low health literacy on patients’ health outcomes: a multicenter cohort study. BMC Health Serv Res. 2022;22(1):1148. 36096793 10.1186/s12913-022-08527-9 PMC9465902

[45] Magallón-Botaya R, et al. Effectiveness of health literacy interventions on anxious and depressive symptomatology in primary health care: A systematic review and meta-analysis. Front Public Health. 2023;11:1007238. 36844856 10.3389/fpubh.2023.1007238 PMC9948257

[46] Holloway I. Qualitative research in health care. McGraw-Hill Education (UK); 2005.

[47] Seale C, et al. Qualitative research practice. Sage; 2004.

[48] Gale NK, et al. Using the framework method for the analysis of qualitative data in multi-disciplinary health research. BMC Med Res Methodol. 2013;13(1):1–8. 24047204 10.1186/1471-2288-13-117 PMC3848812

[49] Tong A, Sainsbury P, Craig J. Consolidated criteria for reporting qualitative research (COREQ): a 32-item checklist for interviews and focus groups. Int J Qual Health Care. 2007;19(6):349–57. 17872937 10.1093/intqhc/mzm042

[50] O’Brien BC, et al. Standards for reporting qualitative research: a synthesis of recommendations. Acad Med. 2014;89(9):1245–51. 24979285 10.1097/ACM.0000000000000388

[51] Cawthon C, et al. Implementing routine health literacy assessment in hospital and primary care patients. Joint Comm J Qual Patient Saf. 2014;40(2):68–AP1. 10.1016/s1553-7250(14)40008-4 PMC4072202 24716329

[52] Boulos MNK. Using social media for improving health literacy. Copenhagen, Denmark: World Health Organization Regional Office For Europe; 2012.

[53] Peterson EB, et al. The role and impact of health literacy on peer-to-peer health communication. Inform Serv Use. 2019;39(1–2):37–49. 10.3233/SHTI200058 32594017

[54] Wittenberg E, et al. Promoting improved family caregiver health literacy: evaluation of caregiver communication resources. Psycho-oncology. 2017;26(7):935–42. 26990206 10.1002/pon.4117 PMC5026538

[55] Barutcu CD. Relationship between caregiver health literacy and caregiver burden. P R Health Sci J. 2019;38(3). 31536629

[56] Meherali S, Punjani NS, Mevawala A. Health literacy interventions to improve health outcomes in low-and middle-income countries. HLRP: Health Lit Res Pract. 2020;4(4):e251–66. 33313935 10.3928/24748307-20201118-01 PMC7751448

[57] Kaper MS, et al. Outcomes and critical factors for successful implementation of organizational health literacy interventions: a scoping review. Int J Environ Res Public Health. 2021;18(22):11906. 34831658 10.3390/ijerph182211906 PMC8622809

[58] Ayre J, et al. Systematic review of health literacy champions: who, what and how? Health Promot Int. 2023;38(4):daad074. 37470429 10.1093/heapro/daad074 PMC10357937

[59] Madden M, Tupper J. Become a health literacy champion: strategies to promote health literacy in athletic training. J Athl Train. 2024;59(5):428. 38243730 10.4085/1062-6050-0390.23 PMC11127681

[60] Gibson C, Smith D, Morrison AK. Improving health literacy knowledge, behaviors, and confidence with interactive training. Volume 6. HLRP: Health Literacy Research and Practice; 2022. pp. e113–20. 2. 10.3928/24748307-20220420-01 PMC9126053 35522855

[61] Rubinelli S, Schulz PJ, Nakamoto K. Health literacy and the tailoring of health information. a dialogue between communication and (AI) technology. In AAAI Fall Symposium: Virtual Healthcare Interaction; 2009.

[62] Adegboye M. Impact of artificial intelligence on health information literacy: guidance for healthcare professionals. Libr Hi Tech News. 2024;41(7):1–5.

[63] Geanta M, et al. Personalized medicine literacy. In Precision medicine in clinical practice. Springer; 2022. pp. 197–219.

[64] Koonce TY, et al. A personalized approach to deliver health care information to diabetic patients in community care clinics. J Med Libr Association: JMLA. 2015;103(3):123. 10.3163/1536-5050.103.3.004 PMC4511051 26213503

[65] Osborne H. Health literacy: how visuals can help tell the healthcare story. J Vis Commun Med. 2006;29(1):28–32. 16766310 10.1080/01405110600772830

[66] Beck T, Giese S, Khoo TK. Harnessing the power of empathy, visual Art and patient narratives to improve health literacy: an exploratory study. Health Promotion J Australia. 2025;36(1):e893. 10.1002/hpja.893 PMC11729824 38951015

[67] Šulinskaitė K, Zagurskienė D, Blaževičienė A. Patients’ health literacy and health behaviour assessment in primary health care: evidence from a cross-sectional survey. BMC Prim Care. 2022;23(1):223. 36064351 10.1186/s12875-022-01809-5 PMC9446736

[68] Singleton K, Krause E. Understanding cultural and linguistic barriers to health literacy. Online J Issues Nurs. 2009;14(3). 21053716

[69] Park S, Lee H, Kang M. Factors affecting health literacy among immigrants-systematic review. Eur J Pub Health. 2018;28(suppl4):cky214.

[70] Ugas M, et al. Associations of health literacy and health outcomes among populations with limited Language proficiency: a scoping review. J Health Care Poor Underserved. 2023;34(2):731–57. 37464529 10.1353/hpu.2023.0039

[71] Wang L. Analysing Spatial accessibility to health care: a case study of access by different immigrant groups to primary care physicians in Toronto. Ann GIS. 2011;17(4):237–51.

[72] Li H, et al. Comparison of perceived quality amongst migrant and local patients using primary health care delivered by community health centres in Shenzhen, China. BMC Fam Pract. 2014;15:1–7. 24779564 10.1186/1471-2296-15-76 PMC4012177

[73] Johnson RM, et al. A novel approach to improve health literacy in immigrant communities. HLRP: Health Lit Res Pract. 2019;3(3):S15–24. 31687655 10.3928/24748307-20190408-01 PMC6826755

[74] Fernández-Gutiérrez M, et al. Health literacy interventions for immigrant populations: a systematic review. Int Nurs Rev. 2018;65(1):54–64. 28449363 10.1111/inr.12373

[75] Morrison AK, et al. Low caregiver health literacy is associated with higher pediatric emergency department use and nonurgent visits. Acad Pediatr. 2014;14(3):309–14. 24767784 10.1016/j.acap.2014.01.004 PMC4003496

