# Supplementary material for: Development of a health literacy framework for primary health care service users in Qatar: a mixed methods study
Source: BMC Health Serv Res. 2026 Jan 2;26:35. doi: 10.1186/s12913-025-13919-8 (PMC12784517; doi:10.1186/s12913-025-13919-8)
Supplement: Supplementary file 1 — Supplementary Material 1 [file 12913_2025_13919_MOESM1_ESM.docx]

**SUPPLEMENTARY FILE**

This appendix has been provided by the authors to give readers additional information about their study.

Supplement to: Development of a health literacy framework for primary health care service users in Qatar: A mixed methods study.

**Table S1: Defining HLQ domains and the high level of each construct**

| **HLQ domain** | **Domain description** | **No of items** | **Response scale** |
| --- | --- | --- | --- |
|  | **HLQ Part 1** |  | 1=strongly disagree; 2=disagree; 3=agree; 4=strongly agree |
| 1 | Feeling understood and supported by healthcare providers | 4 |  |
|  | **High level of the construct:** Have an established relationship with at least one healthcare provider who knows them well and who they trust to provide useful advice and information and to assist them to understand information and make decisions about their health |  |  |
|  |  |  |  |
| 2 | Having sufficient information to manage my health | 4 |  |
|  | **High level of the construct:** Feel confident that they have all the information that they need to live with and manage their condition and to make decisions |  |  |
|  |  |  |  |
| 3 | Actively managing my health | 5 |  |
|  | **High level of the construct:** Recognize the importance and are able to take responsibility for their own health. They proactively engage in their own care and make their own decisions about their health. They make health a priority |  |  |
|  |  |  |  |
| 4 | Social support for health | 5 |  |
|  | **High level of the construct:** A person’s social system provides them with all the support they want or need for health |  |  |
|  |  |  |  |
| 5 | Appraisal of health information | 5 |  |
|  | **High level of the construct:** Able to identify good information and reliable sources of information. They can resolve conflicting information by themselves or with help from others |  |  |
|  |  |  |  |
|  | **HLQ part 2** |  | 1=cannot do or always difficult; 2=usually difficult; 3=sometimes difficult; 4=usually easy; 5=always easy |
| 6 | Ability to actively engage with healthcare providers | 5 |  |
|  | **High level of the construct:** Are proactive about their health and feel in control in relationships with healthcare providers. Are able to seek advice from additional healthcare providers when necessary. They keep going until they get what they want. Empowered |  |  |
|  |  |  |  |
| 7 | Navigating the healthcare system | 6 |  |
|  | **High level of the construct:** Able to find out about services and supports so they get all their needs met. Able to advocate on their own behalf at the system and service level |  |  |
|  |  |  |  |
| 8 | Ability to find good health information | 5 |  |
|  | **High level of the construct:** Are ‘information explorer’. Actively use a diverse range of sources to find information and are up to date |  |  |
|  |  |  |  |
| 9 | Understanding health information well enough to know what to do | 5 |  |
|  | **High level of the construct:** Are able to understand all written information (including numerical information) in relation to their health and able to write appropriately on forms where required |  |  |

Table S2: High-level health literacy rates amongst primary health care service users

|  |  | **Arabic language (N=1420)** | | | **English language (N=1704)** | | | **Overall (Total N=3124)** | | |
| --- | --- | --- | --- | --- | --- | --- | --- | --- | --- | --- |
|  | **High level of the HL construct** | **N** | **%** | **95% CI** | **N** | **%** | **95% CI** | **N** | **%** | **95% CI** |
| Domain 1 | Have an established relationship with at least one healthcare provider who knows them well and who they trust to provide useful advice and information about their health | 428 | 30.1 | (27.8 - 32.6) | 464 | 27.2 | (25.2 - 29.4) | 892 | 28.6 | (27 - 30.2) |
| Domain 2 | Feel confident that they have all the information that they need to live with and manage their condition and to make decisions | 536 | 37.7 | (35.3 - 40.3) | 573 | 33.6 | (31.4 - 35.9) | 1109 | 35.5 | (33.8 - 37.2) |
| Domain 3 | Recognize the importance and are able to take responsibility for their own health. They proactively engage in their own care and make their own decisions about their health. They make health a priority | 594 | 41.8 | (39.3 - 44.4) | 659 | 38.7 | (36.4 - 41) | 1253 | 40.1 | (38.4 - 41.8) |
| Domain 4 | A person’s social system provides them with all the support they want or need for health | 557 | 39.2 | (36.7 - 41.8) | 565 | 33.2 | (31 - 35.4) | 1122 | 35.9 | (34.2 - 37.6) |
| Domain 5 | Able to identify good information and reliable sources of information. They can resolve conflicting information by themselves or with help from others | 637 | 44.9 | (42.3 - 47.5) | 536 | 31.5 | (29.3 - 33.7) | 1173 | 37.5 | (35.9 - 39.3) |
| Domain 6 | Are proactive about their health and feel in control in relationships with healthcare providers and are able to seek advice until they get what they wan (Empowered) | 575 | 40.5 | (38 - 43.1) | 483 | 28.3 | (26.2 - 30.5) | 1058 | 33.9 | (32.2 - 35.5) |
| Domain 7 | Able to find out about services and supports so they get all their needs met. Able to advocate on their own behalf at the system and service level | 476 | 33.5 | (31.1 - 36) | 382 | 22.4 | (20.5 - 24.4) | 858 | 27.5 | (25.9 - 29.1) |
| Domain 8 | Are ‘information explorer’. Actively use a diverse range of sources to find information and are up to date | 491 | 34.6 | (32.1 - 37.1) | 429 | 25.2 | (23.2 - 27.3) | 920 | 29.4 | (27.9 - 31.1) |
| Domain 9 | Are able to understand all written information (including numerical information) in relation to their health and able to write appropriately on forms where required | 676 | 47.6 | (45 - 50.2) | 658 | 38.6 | (36.3 - 40.9) | 1334 | 42.7 | (41 - 44.4) |

**All the differences were statistically significant at 0.05 level of significance because of large sample size.*

**Appendix 1: Focus Group Discussion Guide**

**Broad Theme 1: Understanding Health Literacy**

Sub-Theme: Definitions and Perceptions

- How would you define health literacy in your own words?
- Can you share any experiences that shaped your understanding of health information?

Sub-Theme: Importance of Health Literacy

- In what ways do you believe health literacy affects your general well-being of service users accessing primary care services at PHCC?
- How do you think health literacy impacts the management of chronic conditions?

**Broad Theme 2: Health Management and Chronic Diseases**

Sub-Theme: Experience of Managing Chronic Diseases

- Can you describe your experience with managing patients with chronic conditions?
- What role does health information play in management such patients?

Sub-Theme: Barriers to Effective Management

- What challenges do you feel the services users might face in understanding health information regarding your condition?
- Are there any specific aspects of chronic condition that service users might find difficult to manage due to a lack of information?

**Broad Theme 3: Preferred Health Literacy Channels**

Sub-Theme: Access to Health Information

- What sources of health information are you aware of that are currently used by service users (e.g., healthcare providers, the internet, family, and friends)?
- In your opinion how effective are these sources in providing service users with reliable health information?

Sub-Theme: Channel Preferences

- What formats of health information do you prefer (e.g., printed materials, online articles, videos, face-to-face consultations)?
- In your opinion what would the service users prefer (the various channels of health literacy) to receive important updates about their health condition?

**Broad Theme 4: Evaluation of Existing Health Literacy Strategies**

Sub-Theme: Awareness of Available Resources

- Are you aware of any health literacy programs or resources offered by the Primary Health Care Corporation?
- How did you learn about these resources, and do you find them helpful?

Sub-Theme: Gaps in Current Strategies

- What improvements do you think can be made to existing health literacy strategies in your primary health clinic?
- Are there specific topics or areas where you feel additional information is needed?

**Broad Theme 5: Engagement and Support**

Sub-Theme: Support from Healthcare Providers

- In your experience, what is the role of healthcare providers like yourself to support the understanding health condition of service users accessing primary care services at PHCC?

Sub-Theme: Community and Peer Support

- Do you believe peer support can enhance the understanding of health information of service users? If yes, how?
- Are there community resources you think could help improve health literacy among patients with chronic diseases?

**Conclusion**

- We covered important themes in this FGD including your understanding of the concept of health literacy, your experiences managing chronic diseases, preferred health information channels, and your thoughts on existing health literacy strategies provided by primary health clinics.
- Is there anything that you would like to add which wasn’t discussed or covered in the FGD.
- Thank you for your participation and sharing your invaluable insight.
